# Effective Collaboration Through Activity Theory and Knotworking in Clinical Settings

**DOI:** 10.7759/cureus.19860

**Published:** 2021-11-24

**Authors:** Marvin Mnaymneh, Roland van Oostveen, Bill Kapralos, Adam Dubrowski

**Affiliations:** 1 Education, Ontario Tech University, Oshawa, CAN; 2 Simulation, Software Informatics Research Centre, Ontario Tech University, Oshawa, CAN; 3 Research and Development, Ontario Tech University, Oshawa, CAN

**Keywords:** leadership, activity systems, knotworking, collaboration, activity theory

## Abstract

Healthcare professionals must be able to work in multidisciplinary teams (MDTs). The purpose of this editorial is to explain how healthcare professionals (can) contribute to the effectiveness of MDT, through the use of activity theory (collective work activity shared by others who are motivated by a purpose mediated by tools in order to achieve a specific goal) and the associated idea of knotworking (method of tying, untying, and retying together seemingly separate threads of activity). The leading thesis here is that MDTs benefit from health professionals with well-established leadership skills, and also strong collaborative skills that enable them to transition fluidly between leadership roles as needed to advance patient care. Within activity theory, knotworking is the process of tying and untying various threads of activity and knowledge from across the MDT in order to accomplish specific objectives over time. Knotworking exemplifies the dynamic nature of MDT collaboration, which requires professionals to be productive in their environment. The viewpoints offered in this editorial contribute to a new perspective on MDTs, one that acknowledges distributed leadership and the importance of co-producing a successful partnership in a clinical setting.

## Editorial

Introduction

Within an interprofessional healthcare setting, it is expected that effective collaboration exists between health professionals across an education continuum; however, this proves to be challenging [1]. Multidisciplinary teams (MDTs) are constantly engaged in distributed and unstructured collaboration efforts, using different equipment with different patients, ad hoc rotations, synchronously and asynchronously. The notion that MDTs can always follow a single predefined leader is archaic and counterproductive in light of these challenges. In addition, MDTs are categorized according to hierarchical rank, where the physician be ultimately responsible for patient care [2]. Facing this intimidation is challenging for all MDTs trying to work in a high-paced collaboration environment. The attempt to train MDTs for the rigours of working together will be hampered unless certain issues are addressed. 

The editorial was inspired by an illustrative, fictional scenario-based design using an MDT collaborative approach as an opportunity to study how participants in an MDT context work with and adapt to change in practice. The scenario is presented within a commonplace Obstetrics and Gynaecology clinical setting where MDTs often collaborate and will subsequently illustrate how activity theory and its concept of knotworking are applied within Obstetrics and Gynaecology.

Illustrative scenario-based design

*Phase 1:*Sheila, a 30-year-old woman who is 38 weeks pregnant, has been admitted to the labour and delivery unit, where her partner will join her. Labour began 10 hours ago and has progressed slowly to an 8-cm dilation. Sheila has been cared for by two nurses, one registered nurse (RN), and one registered practical nurse (RPN) since their shift began, taking over from their colleagues two hours earlier. In the last two hours, there has been no increase in dilation. Every time the patient experiences a contraction, the fetal heart rate drops to 90 beats per minute. Between contractions, the average heartbeat of a baby increases from 140 beats per minute to 160 beats per minute. The RN decides to contact the nearest obstetrics resident to discuss the matter.

*Phase 2: *One available resident decides to enter Sheila's room, introduces herself and immediately begins monitoring the baby's fetal heartbeat. The resident says that she will need to measure the amount of dilation present and the location of the baby's head before proceeding. Sheila has given her consent to be examined. The resident begins her examination with the support of the RN, while the RPN monitors Sheila's vital signs. 

*Phase 3:*Due to the baby's head having dropped significantly and dilation remaining at 8 cm, the RN believes that the fetus is in a stable condition. To increase the speed of dilation, the RN recommends that Sheila be given a light sedative to help her contract more quickly. The RPN attempts to join the conversation but is interrupted by the resident, who expresses concerns to the RN about the baby's condition and mentions alternate delivery procedures. In light of the exam, the resident believes that dilation is closer to 7 cm rather than 8 cm and that the baby's head is still elevated. Because Sheila is exhausted and in discomfort during her pregnancy, Sheila asks for an epidural to deliver the baby through vaginal birth. To make the final choice, the resident requests that the RPN page the on-call gynaecologist.

*Phase 4:*In the unit, after completing patient rounds, the gynaecologist makes their way into the unit 15 minutes later. After introducing herself to Sheila and the resident in the room and dismissing the RN and RPN, the gynaecologist begins to probe the resident to describe what she would do if she were the gynaecologist on call to the situation described above. In response to the gynaecologist's question, the resident expresses hesitation about whether a caesarian section is the best option without providing any explanation for her choice. The gynaecologist recommended a caesarian section and agrees with the resident because it is the quickest method of giving birth to the fetus. The gynaecologist also acknowledges the patient's fatigue and desire for a quick resolution, noting that the anaesthesia administered during the procedure will help alleviate the patient's pain.

Thematic Analysis Review Using Activity Theory

Activity theory studies how people's activities impact natural and social reality in complex social interactions [3]. Formerly known as Cultural Historical Activity Theory (CHAT) [3], people and their behaviours can be understood when weighed in the context of social and historical factors shaping those activities [3]. Furthermore, these contexts are constantly changing as a result of the actions of persons inside the environment. In other words, individuals acquire the social and historical conditions in which they find themselves and, at the same time, actively construct, modify, and revise those environments [3]. Activity theory is used to describe actions in a socio-technical system through six related elements [4] of a conceptual activity system influencing human activity: 

(a) Object-orientedness: the objective of the activity system.

(b) Subject or internalization: the actors engaged in the activities. 

(c) Community or externalization: all actors involved in the activity system.

(d) Tools or tool mediation: the collection of objects (or concepts) employed by actors in a system (both material and abstract artefacts).

(e) Division of labour: the division of a work process into a number of tasks, each of which is completed by a distinct individual or group of individuals.

(f) Rules: the guidelines governing the operations of the system as a whole.

Activity systems call attention to the tensions and contradictions inherent in human behaviours and the solutions that can be found to resolve these conflicts and inconsistencies [4]. For example, according to Engeström (2021), looking at medical education via a CHAT lens directs researchers' attention to the ambiguity and surprises that are inherent in human behaviour, in addition, interpretations, and tensions, change and the opportunity for sensemaking [3].

**Figure 1:**
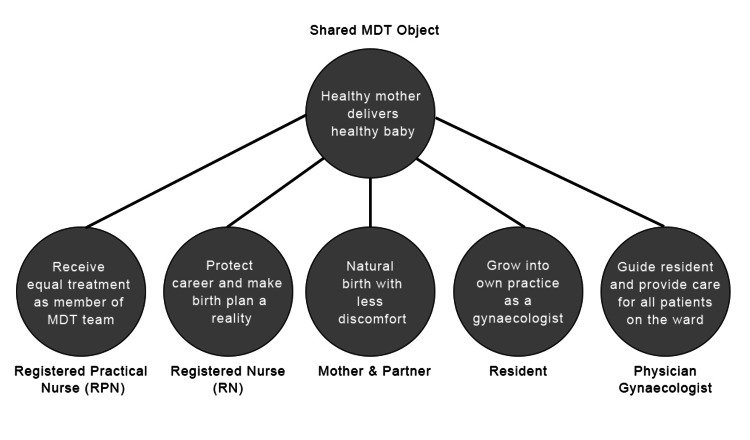
Numerous activity systems with the common goal of delivering a healthy baby.

In the illustrative scenario, multiple personnel were observed caring for a labouring patient. Everyone in the unit (including the patient, the partner, the RN, the RPN, the resident, and the on-call gynaecologist) is working toward the same shared goal: ensuring a healthy baby and mother throughout the delivery (Figure 1). In the meantime, there were conflicts between each collaborator in the shared activity, which caused the shared goal to be put under strain. Sheila, for example, desired a vaginal delivery with the least amount of discomfort. Despite the RN's support for the mother's decision to give birth vaginally, she was concerned about her ability to integrate into the hospital's community and safeguard her profession's reputation in the medical community. The resident desired to become self-sufficient exhibiting her clinical knowledge allowing for her to take on responsibilities of a gynaecologist after completing her residency. The gynaecologist must instruct the resident while also adhering to hospital policies and procedures to provide outstanding patient care to the other labouring moms on the ward. To be considered an equal and a member of the MDTs, the RPN wished to be acknowledged. It is clear in the scenario that culturally, socially, and historically shaped considerations, regulations, and division of labour compliance contributed to the attainment of the shared goal in this scenario.

In summary, activity theory research encourages evaluations of human activities included in MDT practices that are guided by cultural, social, and historical considerations [5]. It enables scholars to investigate the tensions, conflicts, and alignments that exist within and between individual activity systems as well as between these individual activity systems [3].

Knotworking

**Figure 2:**
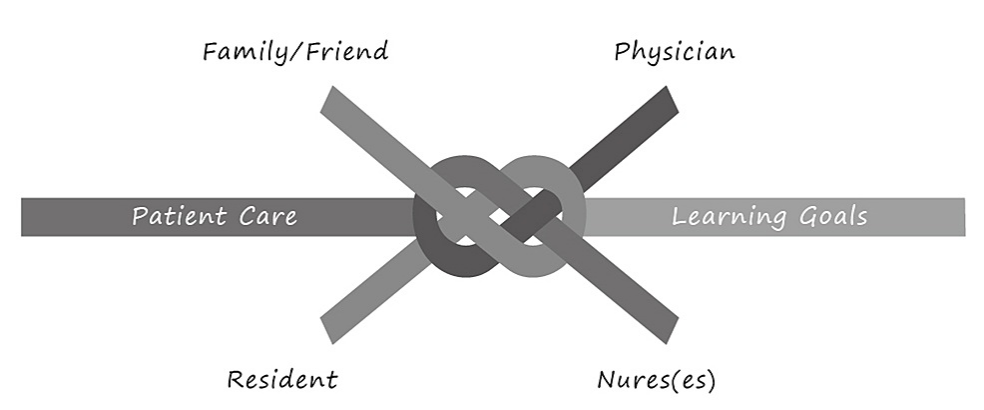
In the context of activity systems, knotworking represents the pieces of a system coming together to perform new activities.

The idea of knotworking was established by Engeström and Pyörälä (2021) to analyze social interactions that occur in a healthcare context that is constantly and dynamically changing. Knotworking has been defined as "a rapidly pulsating, distributed, and largely unplanned orchestration of collaborative performance between otherwise loosely connected actors and activity systems" [3]. According to Engeström and Pyörälä (2021), a knotworking works by untying, retying and tying together various strings of activity and knowledge from across the MDTs to achieve specific objectives (for example, reacting to a patient's need at an appropriate time). The effectiveness of knotworking is dependent on the ability of the collaborators to comprehend one another immediately, coordinate activities effectively, and share control of situations fluidly [5]. As a result, MDTs may find it challenging to manage this last point, which is the sharing of control because it can undermine hierarchies that have historically guided patient care operations. To effectively share patient care delivery/goal, it is necessary for all professions represented in a clinical setting to rethink how power may and should be transferred effectively within the team (Figure 2).

It is necessary to evaluate the collaborative efforts of the MDT as an instance of knotworking to deliver exceptional patient care from three separate dimensions: socio-spatial dimension, temporal dimension, and moral-ideological dimension.

Knotworking: Socio-Spatial Dimension

According to Engeström and Pyörälä (2021), the socio-spatial dimension is concerned with the relationships between activity systems that are active in the knot at any given point in time [3]. Three activity systems actively participate in this knotworking in Phase 4 of the illustrative scenario: the resident, the RN and the RPN, and the gynaecologist. The appraisal of the mother's labour progress was the principal source of strain during the pregnancy.

Knotworking: Temporal Dimension

According to Engeström and Pyörälä (2021), the temporal dimension is one that draws attention to the consecutive steps or episodes in the collaborative endeavour [3]. The illustrative scenario begins 12 hours into the patient's case. Since taking over the mother’s care from the RN and RPN of the previous shift, the current RN and RPN have been on the team for two hours, and a new knot has formed between the labouring mother and the RN and RPN. In Phase 2, the resident adds her thread to the activities formed during Phase 1. In addition, in Phase 4, the gynaecologist joins the MTD, adding their thread to the existing complex knots of relationships.

*Knotworking: Moral*-*Ideological Dimension*

The moral-ideological dimension is included in the final knotworking process, which considers redistribution of power and reconceptualization of control, accountability, and trust required to understand collaborative work [3]. As shown in Phase 1 of the illustrated scenario, the RN assumes the role of leader when she calls the resident to inform them that the patient's condition requires discussion with the MDTs. Phase 2 looks at the resident's thread added to the knot. The leadership is transferred over to the resident, who learned everything about this situation from the RN, begins analyzing the fetal cardiac tracing and determines that a further examination is required. Phase 3, friction or interpersonal conflict between the RN and the resident emerged due to a disagreement about the fetus's condition and what is the best course of action. Phase 4, the gynaecologist enters the knot, tying her thread of activity to the knot and elevating her to the team leader position. Throughout this scenario, the activity knot is changing and shifting, with new collaborators entering the knot and the MDT leadership becoming unclear over time. The ever-changing moral-ideological dimension of MDT collaboration, as well as the unstable allocation of authority and control within the team, must be understood to comprehend MDT collaboration. 

A classic example of knotworking is the MDT collaboration, which has been highlighted in this illustrative scenario. When it comes to MDTs, there is no one point of control; instead, the team's ability to collaborate is dependent on each team member's ability to comprehend and work within the social-spatial, temporal, and moral-ideological dimensions of the knot [5].

Conclusion

To maximize the potential of MDTs in bringing about educational change, we must have a better knowledge of how change occurs in clinical settings and how health professionals apply what they learn in practice. By focusing on activity theory, this editorial aimed to show that practice changes and outcomes realized knotworking collaboration between health professionals in MDTs and their workplaces. Health professionals engaging in difficult or challenging processes on their own should have the assistance and guidance provided to them as a component of their MDT-related learning outcomes. Health professionals in their workplaces must acknowledge that their knowledge will evolve through knotwork collaboration with their colleagues, reconceptualization, and reconciliation. Recognizing the complexities of these processes can work to promote from within, ensuring that MDT collaboration contributes fully to educational reform in practice.
